# Metazoan mitochondrial gene sequence reference datasets for taxonomic assignment of environmental samples

**DOI:** 10.1038/sdata.2017.27

**Published:** 2017-03-14

**Authors:** Ryuji J. Machida, Matthieu Leray, Shian-Lei Ho, Nancy Knowlton

**Affiliations:** 1 Biodiversity Research Centre, Academia Sinica, Taipei 11529, Taiwan; 2 Smithsonian Tropical Research Institute, Panama City, Republic of Panama; 3 National Museum of Natural History, Smithsonian Institution, Washington DC 20560-0163, USA

**Keywords:** Genetic databases, Biodiversity, Mitochondrial genome, Bioinformatics, Classification and taxonomy

## Abstract

Mitochondrial-encoded genes are increasingly targeted in studies using high-throughput sequencing approaches for characterizing metazoan communities from environmental samples (e.g., plankton, meiofauna, filtered water). Yet, unlike nuclear ribosomal RNA markers, there is to date no high-quality reference dataset available for taxonomic assignments. Here, we retrieved all metazoan mitochondrial gene sequences from GenBank, and then quality filtered and formatted the datasets for taxonomic assignments using taxonomic assignment tools. The reference datasets—‘Midori references’—are available for download at www.reference-midori.info. Two versions are provided: (I) Midori-UNIQUE that contains all unique haplotypes associated with each species and (II) Midori-LONGEST that contains a single sequence, the longest, for each species. Overall, the mitochondrial Cytochrome oxidase subunit I gene was the most sequence-rich gene. However, sequences of the mitochondrial large ribosomal subunit RNA and Cytochrome b apoenzyme genes were observed for a large number of species in some phyla. The Midori reference is compatible with some taxonomic assignment software. Therefore, automated high-throughput sequence taxonomic assignments can be particularly effective using these datasets.

## Background & Summary

Massively parallel sequencing technologies have revolutionized our ability to survey and monitor biological diversity. Samples containing multiple species are collected directly from the environment (e.g., from water, sediments, traps, feces) and variants of one or several sets of genes are inventoried using PCR-based (e.g., metagenetics) or PCR-free (e.g., mitogenomics, metatranscriptomics, metagenomics) approaches.

Two types of gene sequences have been widely used as phylogenetic and taxonomic markers in metazoans: nuclear-encoded ribosomal RNA genes (18S and 28S ribosomal RNA genes^1,2^) and mitochondrial-encoded genes^3,4^. Nuclear-encoded ribosomal RNA fragments, especially hypervariable regions of the 18S rRNA gene, were prime targets in early metagenetic analyses because broad-range primers well conserved across the eukaryotic domain were available^2,5,6^. As a result, considerable efforts have already been made to build quality filtered and formatted reference sequence datasets of nuclear-encoded ribosomal RNA genes for taxonomic assignments^7,8^. Mitochondrial genes, which provide higher taxonomic resolution for most metazoan groups^9^, have been increasingly used following the design of highly degenerate primer sets^4,10–12^ and the development of bioinformatics tools to facilitate the assembly of mitogenomes from environmental samples^13^. However, high quality reference datasets that are compatible with taxonomic assignment software are not yet available for metazoan mitochondrial genes. Therefore, at the moment, most of the metazoan metagenetic studies target low-resolution nuclear ribosomal RNA gene as a marker (e.g., SILVA^7^). Some exceptions, which target high-resolution mitochondrial genes, used Blastn searches against sequences from GenBank for taxonomic assignments^10,12^ without explicit taxonomic quality control of the database. This means that high-throughput sequence taxonomic assignment with quality controlled mitochondrial gene reference dataset is currently not feasible. Here, we constructed quality-controlled reference datasets ‘Midori’ for thirteen protein-coding and two ribosomal RNA genes sequences encoded in the mitochondrial genome.

**Figure 1:**
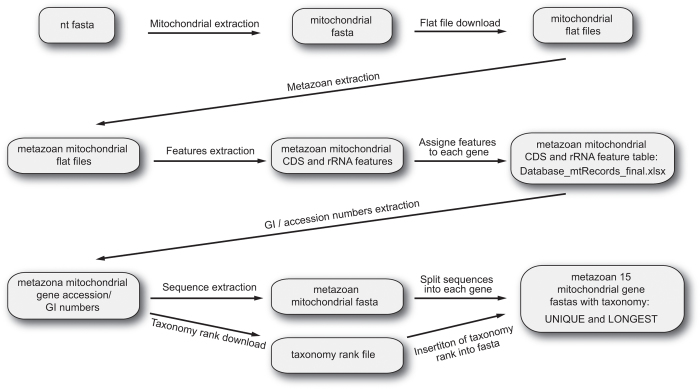


After downloading the nt datasets from GenBank, we implemented the following informatics procedures to construct the datasets (Fig. 1): (I) extraction of mitochondria-related gene sequences, (II) extraction of metazoan gene sequences, (III) insertion of taxonomic ranking information onto each sequence, (IV) removal of sequences that did not have species-level taxonomic information, (V) splitting the sequences into thirteen protein and two ribosomal RNA gene sequences, (VI) preparation of a taxonomy rank file.

**Figure 2:**
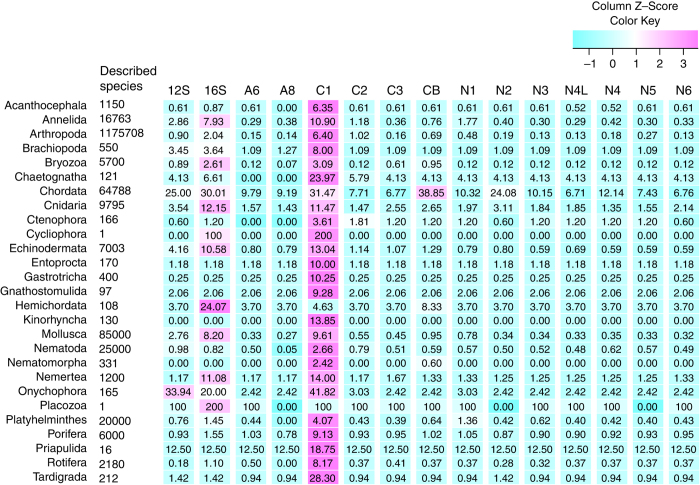
A heat map of observed gene sequence number (*Z*-score transformed percentage) per phylum. Midori-LONGEST (one sequence per species) was used for this comparison. Percentages of species with sequence data are indicated in each column. Loricifera was removed because no mitochondrial sequence was found in the nt dataset. Refer to the text for the abbreviation of gene names.

We have prepared two versions of the 15 datasets: (I) Midori-UNIQUE, which contains for each species a representative sequence of each unique haplotype and (II) Midori-LONGEST, which contains for each species the single longest sequence. Each dataset is composed of two ribosomal RNA (Large [*lrRNA*] and Small [*srRNA*] ribosomal subunit RNA) and thirteen protein (ATP synthase subunit 6 [*A6*] and 8 [*A8*]; Cytochrome oxidase subunit I [*COI*], II [*COII*] and III [*COIII*]; Cytochrome b apoenzyme [*Cytb*]; NADH dehydrogenase subunits 1–4 [*ND1*-*ND4*], 4 L [*ND4L*], 5 [*ND5*] and 6 [*ND6*]) gene sequences (thirteen mitochondrial protein-coding genes found in most but not all phyla of metazoans^14^). In both datasets, *COI* had the largest number of sequences overall (Table 1). However, *Cytb* had a higher number of sequences in Chordata, while the *lrRNA* gene had the highest coverage among Cnidaria, Hemichordata and Placozoa (Fig. 2, Table 2).

We provide two formats of Midori-UNIQUE and Midori-LONGEST (www.reference-midori.info) that are compatible with taxonomic assignment software such as RDP Classifier^15^, SPINGO^16^ and BLAST+ ref. 17. We believe these datasets will improve the accuracy of taxonomic assignments of the massive numbers of sequences obtained from high throughput sequencing experiments.

## Methods

### Mitochondrial DNA sequence curation

The nt fasta file was downloaded from the National Centre for Biotechnology Information (NCBI) server (ftp://ftp.ncbi.nih.gov/blast/db/FASTA) on 18 September 2015 (Fig. 1). Mitochondria-related gene sequences (including nuclear-encoded mitochondrial genes) were extracted from the fasta file using a custom Perl script (02_ext_seq_mito.pl) available online (www.reference-midori.info/download.php#) along with following scripts described below to build the reference datasets. Next, GenBank flat files of all the mitochondria-related gene sequences were downloaded using NCBI Edirect (efetch -db nucleotide -id gene_id -format gb), and metazoan flat files were extracted using a custom perl script (06_ext_fasta_seq.pl). Next, CDS and rRNA features were extracted from the metazoan flat files using a custom Perl script (09_ext_cds_rna.pl), each combination (feature, gene and product) was counted using MySQL, and the CDS and rRNA feature table (11_Database_mtRecords_final.xlsx) was created. The table with feature combinations was manually examined to assign each gene. Sequences that could not be assigned unambiguously to one of the thirteen protein-coding genes or one of the two ribosomal RNA genes were discarded. Accession number, feature, position, gene, product and gene abbreviations of those assigned sequences were extracted using MySQL. The mitochondrial fasta file that was prepared as described above was partitioned in 15 individual fasta files with sequences of each mitochondrial-encoded gene.

### Taxonomic information curation

We extracted the GI number from the mitochondrial fasta file using a custom perl script (15_ext_gi.pl). Then, taxonomy ID and taxonomy ranking were extracted using the gb_taxonomy_tool (https://github.com/spond/gb_taxonomy_tools). These two output files were combined into a taxonomy file using a custom perl script (19_rdp_train_3.pl). RDP classifier^15^ utilizes only eight taxonomic rankings (Root, Kingdom, Phylum, Class, Order, Family, Genus and Species); therefore, we extracted only those ranks. At this stage, we performed the following quality controls: (I) removal of sequences that did not have species name in the species rank; (II) removal of sequences containing the following text in the taxonomy ranks: ‘cf.’, ‘aff.’, ‘sp.’, ‘environment’, ‘undescribed’, ‘uncultured’, ‘complex’, ‘unclassified’, ‘nom.’, ‘nud.’ and ‘unidentif’ (because these terms indicate uncertainty of species identity); (III) removal of sequences with the following identifiers ‘sp0936BC’, ‘MG98.09’, ‘sp0942A’ and ‘EEG-2007’ (since these are obviously not Latin names). This quality filtration was performed using a combination of Perl, MySQL and Unix commands.

### Insertion of sequences into taxonomy rank file

Each mitochondrial sequence prepared previously was added onto the taxonomy rank file using a custom perl script (23_rdp_train_hash.pl). Each sequence was separated into single files using a custom perl script (25_fna_split.pl), and the target region of each gene was excised and separated into different gene regions using a custom perl script (27_gbk_ext_target.pl). In some cases using RDP Classifier^15^, incorrect taxonomy assignment was observed if the reference contained sequences that were very long or very short. Therefore, we removed sequences outside the following length ranges (nt) for *srRNA*: 200–2,000, *lrRNA*: 100–2,500, *A6*: 100–1,000, *A8*: 100–500, *COI*: 100–2,000, *COII*: 100–1,500, *COIII*: 100–1,300, *Cytb*: 100–1,500, *ND1*: 50–1,200, *ND2*: 150–1,500, *ND3*: 100–600, *ND4*: 150–2,000, *ND4L*: 100–700, *ND5*: 150–2,000 and *ND6*: 150–1,500.

### Creation of taxonomy rank file for RDP classifier

First, we counted the number of occurrences of each taxonomy rank. Next, we extracted the eight taxonomic rankings. Then, the taxonomy rank file was formatted in two steps using two custom Perl scripts (trainset_db_taxid.pl and trainset_db_taxid_parent_2.pl). On some occasions, a conflict of taxonomic names was observed, such as the same genus name for animals in different higher taxonomic groups. Such cases, caused by duplicated taxonomic names above the species level (which are prohibited within the Metazoa but occur through error) caused the taxonomic assignment software to report error messages and abort analysis. In those cases, we made some modifications to the taxonomic name, such as addition of a distinguishing number to one of them (e.g., from *Automolus* to *Automolus01*).

### Code availability

Scripts used for the present reference datasets preparation are freely available from the site www.reference-midori.info/download.php#.

## Data Records

All reference datasets are freely available from the Midori reference web site (www.reference-midori.info) and also Dryad Digital Repository (Data Citation 1). Midori-UNIQUE and Midori-LONGEST (see usage notes for more information) are available in two formats, one compatible with the RDP Classifier^15^ and the other compatible with SPINGO^16^. Both formats are compatible with BLAST+^17^; however, because the RDP format contains taxonomic rank information, we recommend using an RDP formatted file as an input to build the local BLAST+^17^ database (see usage notes for more information on how to build the local database). The numbers of sequences included in the datasets are listed in Table 1.

## Technical Validation

Three kinds of quality filtrations were performed in the present study. First, we included only sequences that have binomial names (genus and species names). Here, we assume that species-level identifications of metazoan are more likely to be performed by well-trained taxonomic specialists, although this step does not ensure the absence of mislabelled sequences, it increases taxonomic accuracy, particularly at higher taxonomic levels. For example, a specimen identified at the species level is more likely to have a correct genus name. Second, we also performed systematic sequence length restrictions by removing extremely long or short gene sequences in the original nucleotide datasets (restriction limits are dependent on the genes, see Methods for details). We observed that sequences at the extremities of the length distribution of each gene were more likely the result of mis-annotations. We also observed that the RDP Classifier consistently provided erroneous sequence taxonomic assignments if very long or very short sequences were included in the reference datasets. Third, we attempted to detect and remove taxonomically mislabelled sequences in the datasets. To do so, we performed a high similarity (99%) clustering of the reference datasets (Table 3) within the typical range of intra-specific variations for mitochondrial-encoded genes. We assumed that, given the low threshold, sequences in each cluster should possess same taxonomic labels at least at the higher phylum-, class- and order-level. The clustering was performed on Midori-UNIQUE using UCLUST^18^ and clusters were flagged if they contained sequences of multiple phyla, classes or orders. To identify which sequence was mislabeled in each flagged cluster, we performed a similarity search using the BLAST server^19^ (blastn with ‘low complexity region filter’ and ‘mask for lookup table only’ function disabled). The distance tree functionality on the BLAST server was used to explore phylogenetic relationships with 100 close matches to each query. Sequences confidently identified as mislabelled were deleted from the datasets. Whenever we could not confidently determine which sequence of the cluster was mislabelled we retained all sequences. Overall, we found that the number of such cases was very low (Table 3) which indicates that the upstream quality filtration was effective (e.g., the inclusion of sequences with binomial names only). We did not attempt to detect mislabelled sequences at lower taxonomic levels, such as family, genus and species in the present study as they may result from a range of evolutionary factors (e.g., incipient species, hybridization, introgression) but also from issues related to classification and systematics (e.g., synonyms, taxonomic confusions, taxonomic revisions).

In some cases, we observed missing taxonomic information, such as class, order or family name (e.g., accession numbers JN392469 and JF760210). In those cases, estimation of statistical support for the missing taxonomic level is not feasible. Therefore, we recommend including all standard levels of taxonomic names in the GenBank taxonomy.

## Usage Notes

Midori reference datasets are compatible with the RDP Classifier^15^. An example of RDP Classifier usage, which required two steps, is as follows. The first step consists of training the reference dataset: $ java -Xmx64g (available memory) -jar /path-to-the-file/classifier.jar train -o /path-to-outfolder/out -s one_of_the_MIDORI_Reference.fasta -t./TaxonomyFile.txt. The second step is the actual taxonomic assignment: $ java -Xmx64g (available memory) -jar /path-to-the-file/classifier.jar classify -t /path-to-the-outfile-from-training/rRNAClassifier.properties -o./outfile_name.txt./query.fasta.

We also prepared the Midori reference datasets in a format compatible with SPINGO^16^. An example of SPINGO usage is as follows: $ spingo -b 100 -k 13 -d /database/ one_of_the_MIDORI_Reference.fasta -i /SPINGO-master/infile/query.fasta>outfile.txt.

Both formats are compatible with BLAST+^17^. An example of usage, which required two steps, is as follows. The first step consists of building a local database: $ makeblastdb -in DB.fasta -dbtype nucl. The second step is the actual search: $ blastn -query example.fasta -db DB.fasta. Refer to http://www.ncbi.nlm.nih.gov/books/NBK279668/ for more detailed information.

All three taxonomic assignment approaches can be used with Midori-UNIQUE and Midori-LONGEST.

## Additional Information

**How to cite this article:** Machida, R. J. *et al.* Metazoan mitochondrial gene sequence reference datasets for taxonomic assignment of environmental samples. *Sci. Data* 4:170027 doi: 10.1038/sdata.2017.27 (2017).

**Publisher’s note:** Springer Nature remains neutral with regard to jurisdictional claims in published maps and institutional affiliations.

## Supplementary Material



## Figures and Tables

**Table 1:** Numbers of sequences included in the reference datasets.

**Gene**	**UNIQUE**	**LONGEST**
*srRNA*	66,937	30,866
*lrRNA*	146,164	54,728
*A6*	23,819	8,947
*A8*	13,187	8,135
*COI*	583,043	110,704
*COII*	44,046	18,320
*COIII*	15,940	7,436
*Cytb*	223,247	35,079
*ND1*	34,090	14,038
*ND2*	72,482	18,880
*ND3*	15,397	9,025
*ND4L*	9,987	6,729
*ND4*	36,819	10,892
*ND5*	28,657	8,793
*ND6*	12,223	6,795
Refer to the text for the abbreviation of gene names.		

**Table 2:** Percentage of species with mitochondrial gene sequences out of the total described or estimated species in each phylum.

		**Gene**														
	**Number of species (described/estimated)**	***srRNA***	***lrRNA***	***A6***	***A8***	***COI***	***COII***	***COIII***	***Cytb***	***ND1***	***ND2***	***ND3***	***ND4L***	***ND4***	***ND5***	***ND6***
Acanthocephala	(1,150/1,500)	0.61/0.47	0.87/0.67	0.61/0.47	0/0	6.35/4.87	0.61/0.47	0.61/0.47	0.61/0.47	0.61/0.47	0.61/0.47	0.61/0.47	0.52/0.40	0.52/0.40	0.61/0.47	0.61/0.47
Annelida	(16,763/30,000)	2.86/1.60	7.93/4.43	0.29/0.16	0.38/0.21	10.90/6.09	1.18/0.66	0.36/0.20	0.76/0.42	1.77/0.99	0.40/0.22	0.30/0.17	0.29/0.16	0.42/0.23	0.30/0.17	0.33/0.18
Arthropoda	(1,175,708/5,892,000)	0.90/0.18	2.04/0.41	0.15/0.03	0.14/0.03	6.40/1.28	1.02/0.20	0.16/0.03	0.69/0.14	0.48/0.09	0.19/0.04	0.13/0.03	0.13/0.03	0.18/0.04	0.27/0.05	0.13/0.03
Brachiopoda	(550)	3.45	3.64	1.09	1.27	8.00	1.09	1.09	1.09	1.09	1.09	1.09	1.09	1.09	1.09	1.09
Bryozoa	(5,700/5,000)	0.89/1.02	2.61/2.98	0.12/0.14	0.07/0.08	3.09/3.52	0.12/0.14	0.61/0.70	0.95/1.08	0.12/0.14	0.12/0.14	0.12/0.14	0.12/0.14	0.12/0.14	0.12/0.14	0.12/0.14
Chaetognatha	(121)	4.13	6.61	0	0	23.97	5.79	4.13	4.13	4.13	4.13	4.13	4.13	4.13	4.13	4.13
Chordata	(64,788/80,500)	25.00/20.12	30.01/24.15	9.79/7.88	9.19/7.40	31.47/25.32	7.71/6.21	6.77/5.45	38.85/31.26	10.32/8.30	24.08/19.38	10.15/8.17	6.71/5.40	12.14/9.77	7.43/5.98	6.76/5.44
Cnidaria	(9,795)	3.54	12.15	1.57	1.43	11.47	1.47	2.55	2.65	1.97	3.11	1.84	1.85	1.35	1.55	2.14
Ctenophora	(166/200)	0.60/0.50	1.20/1.00	0/0	0/0	3.61/3.00	1.81/1.50	1.20/1.00	1.20/1.00	1.20/1.00	0.60/0.50	1.20/1.00	1.20/1.00	1.20/1.00	1.20/1.00	0.60/0.50
Cycliophora	(1)	0	100	0	0	200	0	0	0	0	0	0	0	0	0	0
Echinodermata	(7,003/14,000)	4.16/2.08	10.58/5.29	0.80/0.40	0.79/0.39	13.04/6.52	1.14/0.57	1.07/0.54	1.29/0.64	0.79/0.39	0.80/0.40	0.59/0.29	0.69/0.34	0.59/0.29	0.59/0.29	0.59/0.29
Entoprocta	(170/170)	1.18/1.18	1.18/1.18	1.18/1.18	1.18/1.18	10.00/10.00	1.18/1.18	1.18/1.18	1.18/1.18	1.18/1.18	1.18/1.18	1.18/1.18	1.18/1.18	1.18/1.18	1.18/1.18	1.18/1.18
Gastrotricha	(400)	0.25	0.25	0.25	0.25	10.25	0.25	0.25	0.25	0.25	0.25	0.25	0.25	0.25	0.25	0.25
Gnathostomulida	(97)	2.06	2.06	2.06	2.06	9.28	2.06	2.06	2.06	2.06	2.06	2.06	2.06	2.06	2.06	2.06
Hemichordata	(108/110)	3.70/3.64	24.07/23.64	3.70/3.64	3.70/3.64	4.63/4.55	3.70/3.64	3.70/3.64	8.33/8.18	3.70/3.64	3.70/3.64	3.70/3.64	3.70/3.64	3.70/3.64	3.70/3.64	3.70/3.64
Kinorhyncha	(130)	0	0	0	0	13.85	0	0	0	0	0	0	0	0	0	0
Mollusca	(85,000/200,000)	2.76/1.17	8.20/3.49	0.33/0.14	0.27/0.12	9.61/4.09	0.55/0.23	0.45/0.19	0.95/0.40	0.78/0.33	0.34/0.14	0.34/0.15	0.33/0.14	0.35/0.15	0.33/0.14	0.32/0.14
Nematoda	(25,000/500,000)	0.98/0.05	0.82/0.04	0.50/0.03	0.05/0	2.66/0.13	0.79/0.04	0.51/0.03	0.59/0.03	0.57/0.03	0.50/0.03	0.52/0.03	0.48/0.02	0.62/0.03	0.57/0.03	0.49/0.02
Nematomorpha	(331/2,000)	0/0	0/0	0/0	0/0	2.42/0.40	0/0	0/0	0.60/0.10	0/0	0/0	0/0	0/0	0/0	0/0	0/0
Nemertea	(1,200/10,000)	1.17/0.14	11.08/1.33	1.17/0.14	1.17/0.14	14.00/1.68	1.17/0.14	1.67/0.20	1.33/0.16	1.33/0.16	1.25/0.15	1.25/0.15	1.25/0.15	1.25/0.15	1.25/0.15	1.33/0.16
Onychophora	(165/220)	33.94/25.45	20.00/15.00	2.42/1.82	2.42/1.82	41.82/31.36	3.03/2.27	2.42/1.82	2.42/1.82	3.03/2.27	2.42/1.82	2.42/1.82	2.42/1.82	2.42/1.82	2.42/1.82	2.42/1.82
Placozoa	(1)	100	200	100	0	100	100	100	100	100	0	100	100	100	0	100
Platyhelminthes	(20,000/80,000)	0.76/0.19	1.45/0.36	0.44/0.11	0/0	4.07/1.02	0.43/0.11	0.39/0.10	0.64/0.16	1.36/0.34	0.42/0.10	0.62/0.16	0.40/0.10	0.42/0.11	0.40/0.10	0.43/0.11
Porifera	(6,000/18,000)	0.93/0.31	1.55/0.52	1.03/0.34	0.78/0.26	9.13/3.04	0.93/0.31	0.95/0.32	1.02/0.34	1.05/0.35	0.87/0.29	0.90/0.30	0.90/0.30	0.92/0.31	0.93/0.31	0.95/0.32
Priapulida	(16)	12.50	12.50	12.50	12.50	18.75	12.50	12.50	12.50	12.50	12.50	12.50	12.50	12.50	12.50	12.50
Rotifera	(2,180)	0.18	1.10	0.50	0	8.17	0.37	0.41	0.37	0.37	0.28	0.32	0.37	0.37	0.37	0.37
Tardigrada	(212)	1.42	1.42	0.94	0.94	28.30	0.94	0.94	0.94	0.94	1.42	0.94	0.94	0.94	0.94	0.94
Midori-LONGEST (longest sequence for each species) was used for this calculation. Numbers of described and estimated species follow Chapman^20^; if upper and lower bounds were denoted, we took the upper bound value. The number of Arthropoda species is the combined value of Insecta, Arachnida, Pycnogonida, Myriapoda, Crustacea and Hexapoda. Loricifera was removed because no mitochondrial sequence was found in the nt dataset. Refer to the text for the abbreviation of gene names. Thirteen mitochondrial protein-coding genes found in most but not all phyla of metazoans^13^.																

**Table 3:** Number of clusters containing multiple higher level taxonomic groups (phylum, class, order) after 99% similarity clustering.

**Gene**	**Number of clusters with multiple taxonomic groups**	**Number of removed sequences**		
	**Phylum**	**Class**	**Order**	
*srRNA*	0	5	23	17
*lrRNA*	7	12	44	67
*A6*	0	0	9	9
*A8*	0	0	8	7
*COI*	33	61	210	245
*COII*	0	0	9	16
*COIII*	0	0	8	3
*Cytb*	13	19	66	101
*ND1*	0	1	5	4
*ND2*	0	0	9	8
*ND3*	0	0	6	6
*ND4L*	0	0	4	2
*ND4*	0	0	4	3
*ND5*	0	0	6	6
*ND6*	0	0	3	3
Numbers of problematic sequences removed for this reason are also denoted. Midori-UNIQUE was used for this analysis.				

## References

[d1] Dryad Digital Repository Machida R. J. 2016 http://dx.doi.org/10.5061/dryad.2v00t

